# Acclimation of a low iron adapted *Ostreococcus* strain to iron limitation through cell biomass lowering

**DOI:** 10.1038/s41598-017-00216-6

**Published:** 2017-03-23

**Authors:** Hugo Botebol, Gaelle Lelandais, Christophe Six, Emmanuel Lesuisse, Arnaud Meng, Lucie Bittner, Stéphane Lecrom, Robert Sutak, Jean-Claude Lozano, Philippe Schatt, Valérie Vergé, Stéphane Blain, François-Yves Bouget

**Affiliations:** 1 Sorbonne Universités, Université Pierre et Marie Curie (Paris 06) & Centre National pour la Recherche Scientifique CNRS, UMR 7621, Laboratoire d’Océanographie Microbienne, Observatoire Océanologique, F-66650 Banyuls/mer, France; 2 Université Paris Diderot (Paris 07), Centre National de la Recherche Scientifique, Institut Jacques Monod, F–75013 Paris, France; 3 Sorbonne Universités, Université Pierre et Marie Curie (Paris 06) & Centre National pour la Recherche Scientifique, UMR 7144, Adaptation et Diversité en Milieu Marin, Equipe Marine Phototrophic Prokaryotes, Station Biologique de Roscoff, 29680 Roscoff Cedex, France; 4 Sorbonne Universités, UPMC Univ Paris 06, CNRS, Institut de Biologie Paris-Seine (IBPS), Evolution Paris Seine, F-75005 Paris, France; 5 Department of Parasitology, Faculty of Science, Charles University, 12844 Prague, Czech Republic

## Abstract

Iron is an essential micronutrient involved in many biological processes and is often limiting for primary production in large regions of the World Ocean. Metagenomic and physiological studies have identified clades or ecotypes of marine phytoplankton that are specialized in iron depleted ecological niches. Although less studied, eukaryotic picophytoplankton does contribute significantly to primary production and carbon transfer to higher trophic levels. In particular, metagenomic studies of the green picoalga *Ostreococcus* have revealed the occurrence of two main clades distributed along coast-offshore gradients, suggesting niche partitioning in different nutrient regimes. Here, we present a study of the response to iron limitation of four *Ostreococcus* strains isolated from contrasted environments. Whereas the strains isolated in nutrient-rich waters showed high iron requirements, the oceanic strains could cope with lower iron concentrations. The RCC802 strain, in particular, was able to maintain high growth rate at low iron levels. Together physiological and transcriptomic data indicate that the competitiveness of RCC802 under iron limitation is related to a lowering of iron needs though a reduction of the photosynthetic machinery and of protein content, rather than to cell size reduction. Our results overall suggest that iron is one of the factors driving the differentiation of physiologically specialized *Ostreococcus* strains in the ocean.

## Introduction

Iron is an essential element for all living organisms and in particular for photosynthetic phytoplanktonic cells in which numerous iron-sulphur centre and heme containing proteins are involved in the photosynthetic electron transfer and the nitrate reduction reactions. The extensive use of iron in the cellular machinery of modern microorganisms is inherited from the growth of their ancestors in the iron rich Archean ocean. However the geochemical evolution of the chemical composition of the ocean, largely driven by the evolution of life, has led to vanishing iron concentrations in the present ocean^1^. Thus, iron is a limiting resource in very large regions of the ocean.

Phytoplanktonic species are diverse in size, shape, phylogenetic origin and have evolved specific strategies to colonize ecological niches where iron is limiting (for a review see ref. 2). Physiological and molecular responses to iron limitation have been well studied in nano and microphytoplankton. Cultures of different species have revealed the critical role surface/volume ratio to deal with iron limitation^3, 4^ but several other adaptations to low iron conditions have also been reported. In oceanic diatoms, for example, the lowering of iron needs can be achieved by optimizing the architecture of the photosynthetic machinery through a decrease of the iron-rich complexes such as photosystem I and cytochrome *b*_*6*_/*f* ^5–7^. Iron storage into the ferritin protein pool also constitutes an efficient physiological strategy to cope with sporadic iron supply in pennate diatoms^8^.

The acclimation and adaptation strategies are less understood in picophytoplankton, despite the fact that small phytoplanktonic cells, such as the tiny picocyanobacterium *Prochlorococcus*, numerically dominate oligotrophic water phytoplanktonic communities^9, 10^. Metagenomic and physiological studies in iron depleted regions point to the presence of *Prochlorococcus* clades adapted to iron scarcity^11–13^. These adaptations could notably rely on the selection of specific gene repertoires during the evolution, such as the replacement of iron Super Oxide Dismutase (SOD) by Nickel SOD^14, 15^. Together, genomic and phylogeographic studies strongly suggest the existence of iron adapted lineages in prokaryotic picophytoplankton. A recent study in *Synechococcus* revealed that a coastal strain acclimates to changes in Fe concentration by modulating iron uptake, storage, and photosynthetic proteins^16^.

We recently unveiled a central role for ferritin in the day-night regulation of iron homeostasis in the picoeukaryotic alga *Ostreococcu*s *tauri* and showed that the transcriptional response to iron limitation in this microorganism is tighly dependent on the diurnal cycle^17, 18^. Still, the adaptive responses of eukaryotic picophytoplankton to low iron conditions remain largely unexplored. Within the photosynthetic picoeukaryotes, the order of *Mamelliales* (*Mamiellophyceae*, *Chlorophyta*), encompassing the genera *Ostreococcus*, *Bathycoccus* and *Micromonas*, has a worldwide geographic distribution and it has been shown to contribute significantly to primary production in coastal ecosystems^19–22^. Ribosomal DNA sequence phylogenies segregate *Ostreococcus* sp into 4 phylogenetic clades (A-D) and physiological studies have demonstrated the existence of high- and low-light adapted strains in the genus *Ostreococcus* ^23–26^. Specific primers were successfully developed to amplify Ostreococcus clades A, B and C 18S ribosomal DNA, enabling the study of niche-partitioning in natural populations^27^. Coastal, meso- to eutrophic areas were dominated by clades A and C (renamed clade Ol), whereas open-ocean, oligotrophic regions were dominated by clade B (renamed clade OlI)^27^. This study also indicated that the distribution of *Ostreococcus* lineages is not explained primarily by light irradiance, and that the specialization in different nutrient regimes along coast-offshore gradients might be a key driver of picoeukaryotes evolution/diversification^27^.

In this paper, we compare the physiological responses to iron limitation of several *Ostreococcus* strains inhabiting different environments. Using a combination of physiological and transcriptomic approaches, we unveil several aspects of the specialization of *Ostreococcus* to environments with different levels of iron bioavailability. In particular, we have characterized a Mediterranean strain, RCC802, which shows very low iron requirement, and whose main adaptation trait to iron limitation is a marked cell biomass reduction.

## Material and Methods

### Algal Strains and culture conditions

*Ostreococcus* strains were obtained from the Roscoff Culture Collection (http://www.roscoff-culture-collection.org/): *Ostreococcus tauri* strain OTTH595 (RCC745; isolated from Thau Lagoon, France), *Ostreococcus* sp. RCC802 (isolated at 65 m in the Sicily channel, Italy), *Ostreococcus* sp. RCC809 (isolated from 105 m in the tropical Atlantic Ocean), and *Ostreococcus* sp. RCC789 (strain BL_82-7_clonal, isolated from surface water of Barcelona harbour, Spain).

Cells were grown at 20 °C under 25 µmol quanta m^−2^ s^−1^ of constant blue light (blue led, λ_MAX_ = 465 nm) in AQUIL medium^17, 28^ containing concentrations of Fe(III)-EDTA ranging between 5.4 and 270 nM, corresponding to the theoretical solubility limit of iron in sea water. All culture manipulations were conducted in clean room (class 10,000) equipped with a laminar flow hood (class 100). Synthetic ocean water (SOW) and solutions of inorganic nutrients (NO_3_^−^ and PO_4_^3−^) were separately purified by removing trace metals using a Chelex 100 ion exchange resin (Bio-Rad). Final nutrients concentrations were 300 µM NO_3_^−^ and 10 µM PO_4_^3−^. Trace metal solutions were buffered with 0.1 M of EDTA. For iron content determinations, cells were grown in modified F (Mf) medium^29, 30^ and iron was provided as radioactive ^55^Fe(III)-EDTA (1:20).

### Growth rates and cell diameter measurement

To remove contaminating iron and deplete intracellular iron storage, *Ostreococcus* strains were first acclimated during 5 days in 1.5 mL of AQUIL medium containing 5.4 nM Fe(III)-EDTA (1:1). To study the growth response function of iron supply, triplicate flasks containing various concentrations of Fe(III)-EDTA ranging from 5.4 nM to 270 nM Fe(III)-EDTA in Aquil medium, were inoculated with 10^6^ cells mL^−1^ of iron-depleted cells. These cultures were grown at 20 °C, under 25 µmol quanta m^−2^ s^−1^ of continuous blue light, as described above. Cell density and chlorophyll (Chl) red fluorescence were measured using a flow cytometer (BD Accury C6). Growth rates were computed as the slope of a Ln (Nt) *vs*. time plot, where Nt is the cell abundance (cell/mL) number at time t. Average cell diameter was estimated using a CASY multi-channel cell counting system. (Schärfe System GmbH, Germany).

### Determination of photosynthetic parameters and chlorophyll quantitation

Light-response curves were recorded using a pulse amplitude modulated fluorometer (Phyto-PAM, Walz) connected to a chart recorder (Labpro, Vernier). Chlorophyll contents were determined using HPLC (Hewlett-Packard HPLC 1100 Series). Details are given in Supplementary information online.

### Intracellular iron content measurements

Cells were grown in Mf medium devoid of iron for a week, and were then transferred to Mf medium containing 1 to 100 nM of ^55^Fe (III) EDTA (1:20, 29,600 MBq/mg). The iron cell content was determined by scintillation counting after bleaching photosynthetic pigments with sodium hypochlorite, as previously described^17, 29^.

### Protein extraction and quantitation

The different *Ostreococcus* strains were grown in AQUIL medium containing various concentrations of Fe(III)-EDTA, in 24-wells microplates, under 20 µmol quanta m^−2^ s^−1^ of blue light irradiance at 20 °C. After 6 days, cells were harvested in 2 mL micro-tubes by centrifugation at 8 000 × *g* for 10 min. Dry pellets were frozen in liquid nitrogen ant stored at at −80 °C until extraction. All manipulations were carried out on ice. Cell pellets were ground in 50 µL extraction buffer (50 mM Tris-HCl pH 7.4, 100 mM NaCl, 5 mM EDTA, 0.5% (v/v) Nonidet NP40 (Sigma), 10% (v/v) glycerol, using a Tissue Lyser system (Quiagen). The samples were then centrifuged at 10 000 × *g* for 10 min to remove filter debris. The supernatant was collected, and the total protein concentration was determined using BCA (Bicinchoninic Acid) assay as described elsewhere^31^.

### Oxygen measurements

Oxygen levels were measured using a 24-channel sensor dish oxygen reader (Presens, Regenburg Germany). Cells were grown for one week in sealed OxoDishes® 4 mL vials, in AQUIL medium containing various concentrations of Fe(III)-EDTA. The sensor was placed at 20 °C under blue light. Before recording, light was switched off for 12 hours and the oxygen levels were then measured for 1 h under 20 µmol quanta m^−2^ s^−1^ blue light. Oxygen consumption, *i*.*e*. respiration, was subsequently monitored during 1 hour in darkness. Positive control consisted of cell-free medium oxygenated for 1 h. The negative control (0% O_2_) was obtained by dissolving 0.2 g of sodium L-ascorbate in 0.1 M NaOH.

### RNA extraction and sequencing

Cell cultures were grown for 5 days in 100 mL AQUIL medium containing 5.4 nM (−Fe condition) or 270 nM (+Fe condition) Fe(III)-EDTA. Cultures were placed under 12:12 light/dark cycles of blue light at 40 µmol quanta m^−2^ s^−1^. Three cultures were harvested at 3 h, 9 h, 15 h, 22 h (time 0 h corresponded to dawn and time 12 h to dusk), by centrifugation for 10 min at 8 000 × *g*. Pellets were frozen in liquid nitrogen and stored at −80 °C. RNA extraction was carried out as described by Moulager *et al*.^32^. All manipulations were carried out on ice. The 24 RNA samples (12 +Fe, 12 –Fe) were submitted to high-throughput RNA sequencing using Illumina HiSeq™ 2000 custom pair-end stranded sequencing (Fasteris, Switzerland). After quality controls and filtering of low quality reads (bases quality lower than 15 on sliding window size of 4 bases), clean reads from all samples were gathered, resulting in a dataset of around 300 millions reads. Since there is no reference genome in RCC802, webuild by de novo assembly a reference transcriptome with the TRINITY program in version 2.1.1^33^ using the default parameters and paired-end method. The transcript expression levels in each sample was determined by mapping each read from the sample to the reference transcriptome using BOWTIE^34^ and counting the number of aligned reads using BEDTOOLS^35^. We next applied the DEseq program^36^ to identify differentially expressed genes and calculate an associated risk of error (p-value). Different filtering procedures were finally performed in order to remove potential chimera. Systematic sequence comparisons with *O*. *tauri* (http://bioinformatics.psb.ugent.be/orcae/overview/OsttaV2) coding sequences were performed using tblastx program in version 2.2.29+^37^ with an evalue of 10 e^−30^. The whole procedure is detailed in Supplementary information online.

## Results

### Growth rates of *Ostreococcus* strains under iron limitation

We selected a panel of *Ostreococcus* strains isolated in contrasted trophic regimes and representative of each of the different clades (Table 1). All strains were isolated in the Mediterranean Sea, but the clade B low light ecotype RCC809, which was isolated in the tropical Atlantic Ocean at 105 m depth. Two coastal strains were included in the study, RCC789 (clade D), from Barcelona harbour, and the lagoon strain OTTH595 (clade C) known as *Ostreococcus tauri*, the first *Ostreococcus* species discovered in the *Thau* lagoon in 1995^38^. Both *Ostreococcus tauri* and RCC789 come from eutrophic areas where nutrient bioavailability is high, including iron. RCC802 (clade A) has been isolated at a 65 m depth between Sicily and Tunisia during the PROSOPE cruise^39^. Both RCC802 and RCC809 come from nutrient poor environments that display low Chl *a* concentrations in surface waters (Supplementary Figure 1)^40^. These geographic zones, however, are exposed to sporadic iron fertilization by aeolian mineral dust from the Sahara desert^41, 42^.

**Table 1:** Information regarding the *Ostreococcus* strains used in this study.

**Strain name**	*OTTH595*	*BL*_*82*-*7*_*clonal*	*Eum16BBL*_*clonal*	*PROSOPE*_*44*_*clonal*
**#RCC**	745	789	809	802
**Clade** ^**1**^	C	D	B	A
**Clade** ^**2**^	OI		OII	OI
**Latitude** ^**3**^	+43° 24′	+41° 23′	+21° 2′	+36° 29′
**Longitude** ^**3**^	+3° 36′	+2° 10′	−31° 8′	+13° 19′
**Depth** (**m**)	Surface	Surface	105	65
**Region**	Thau lagoon, France	Barcelona harbour, Spain	Tropical Atlantic Ocean	Sicily channel, Italy
**Trophic level**	Meso/eutrophic	Mesotrophic	Oligotrophic	Oligotrophic

^1^According to refs 24, 49.

^2^According to refs 24, 49.

^3^Localisation on a map in Supplementary Figure 1.

**Figure 1:**
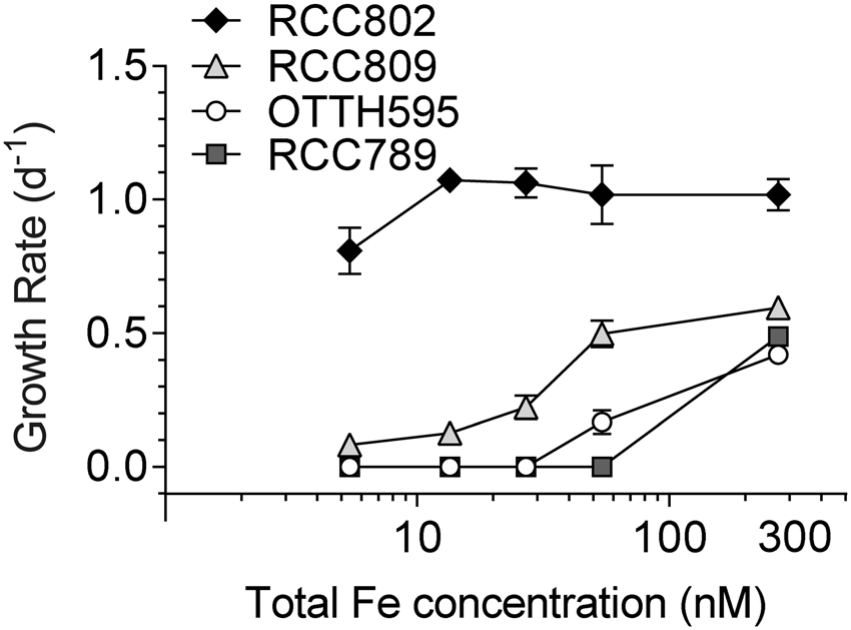
Iron requirements of *Ostreococcus strains*. Growth rates of *Ostreococcus* strains, OTTH595, RCC789, RCC809 and RCC802, were determined in response to various iron supply. Cells were first acclimated for one week in low-iron Aquil medium (5.4 nM Fe(III)-EDTA) before being transferred to Aquil medium containing various concentrations of Fe(III)-EDTA (5.4 nM to 270 nM Fe(III)-EDTA). Growth rates were determined in exponential phase. Mean ± SD of 3 experiments.

*Ostreococcus* strains were acclimated in AQUIL medium containing 5.4 nM Fe(III)-EDTA), to set the cells at the minimum iron level. The growth rates were then measured in response to a supply of various concentrations of total Fe, ranging from 5.4 to 270 nM. Upon transfer to iron replete conditions (270 nM total Fe), RCC802 displayed higher growth rates than the three other strains (Fig. 1). This strain outcompeted all other strains, maintaining high growth rates (∼1 d^−1^) for all Fe concentrations, except for 5.4 nM total Fe concentration, at which a 20% decrease in growth rate (0.8 ± 0.09 d^−1^) was observed. The coastal strains OTTH595 and RCC789 were the most sensitive to iron limitation. Their growth rates dropped dramatically for total Fe concentrations lower than 270 nM (0.42 d^−1^ ± 0.02 at 270 nM to 0.16 d^−1^ ± 0.04 at 54 nM total Fe for OTTH595; no growth of RCC789 from 54 nM). RCC809 displayed an intermediate response with a growth rate of 0.5 d^−1^ and of 0.15 d^−1^ at 54 nM and 5.4 nM total Fe, respectively.

### Photosynthetic parameters of iron limited cells

**Figure 2:**
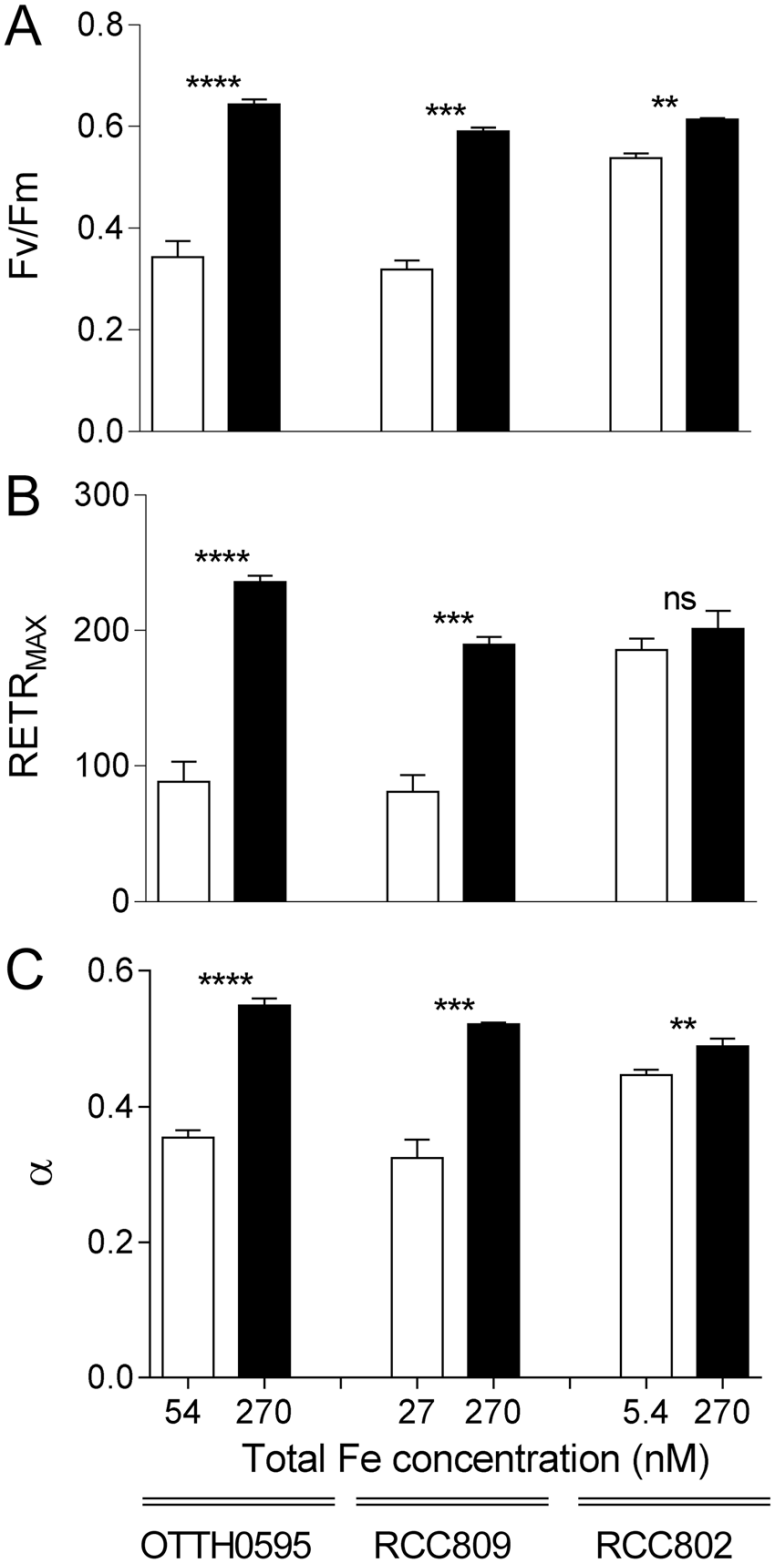
Effect of iron limitation on photosynthetic parameters as derived from light response curves. (**A**) Maximum photosystem II quantum yield of light photoconversion (F_V_/F_M_), measured in the dark, (**B**) Maximal relative Electron Transfer Rate (rETR_MAX_) in photosystem II and (**C**) Initial slope of the light response curve (α), reflecting the functional photosynthetic antenna size. The white boxes correspond to iron limiting concentrations (OTTH595: 54 nM; RCC809: 27 nM; RCC802: 5.4 nM) and the black boxes to iron replete conditions (270 nM). Mean ± SD from 3 experiments.

The efficiency of light energy conversion by photosynthesis was studied using PAM fluorimetry (Fig. 2). Since the coastal strains OTTH595 and RCC789 exhibited very similar responses to iron limitation in terms of growth rates (Fig. 1), only OTTH595 together with RCC802 and RCC809 were further studied. In our standard culture conditions, all strains displayed optimal F_V_/F_M_ values of about 0.65, as reported in previous studies^26^. We compared the photosystem II quantum yield of the three strains in iron replete and limiting conditions, corresponding to the lowest iron concentrations at which cell growth was observed, *i*.*e*. 54, 27 and 5.4 nM of total Fe for OTTH595, RCC809 and RCC802, respectively (Fig. 1).Under iron limitation, the photosystem II quantum yield of OTTH595 and RCC809 strains decreased by about 50% compared to iron replete conditions (from 0.64 to 0.34 for OTTH595 and from 0.59 to 0.31 for RCC809). For RCC802, only a moderate decrease of about 10% (from 0.61 to 0.54) was observed (Fig. 2A).

Light response curves were recorded to evaluate photosynthetic capacities. The maximal relative Electron Transfer Rate (rETR_MAX_) in photosystem II, a key photosynthetic complex located in the thylakoidal membranes of the plastid, dramatically dropped by more than 50% for both OTTH595 and RCC809 respectively. By contrast, no significant change in rETRmax values was observed for RCC802 (Fig. 2B). The α parameter, which corresponds to the initial slope of the light response curve, is related to the light harvesting efficiency of PSII at limiting light irradiance and thus provides indications on the functional size of the photosystem II antenna. Under iron limitation the α parameter of OTTH595 and RCC809 was 35% lower than under replete conditions, indicating reduced light harvesting capacities. As for rETR_MAX_, RCC802 show less than 10% decrease in the α parameter between iron depleted and replete conditions (Fig. 2C).

### Iron content

**Figure 3:**
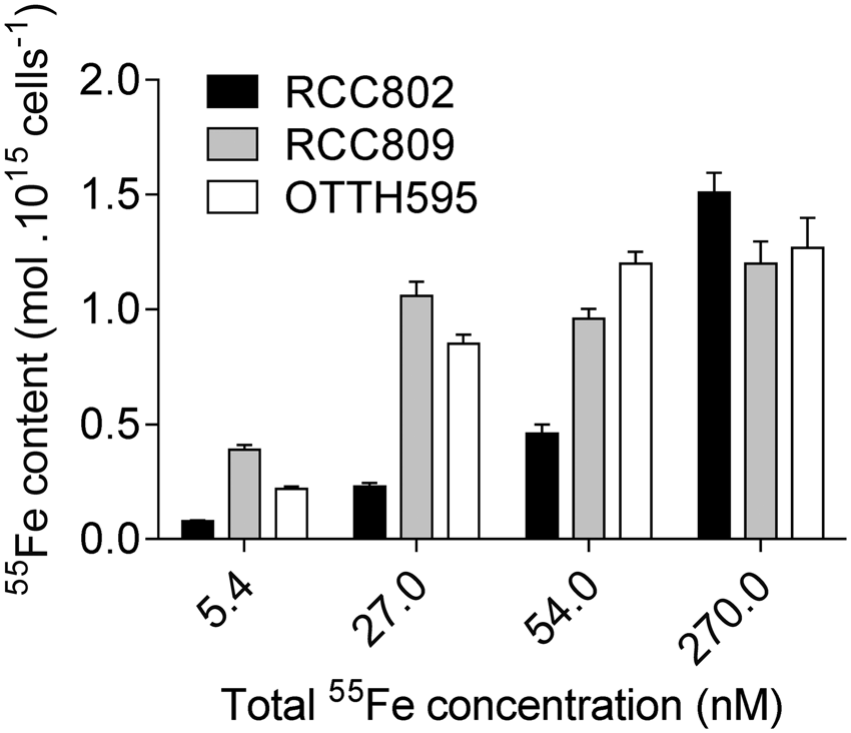
Iron content. *Ostreococcus* cells were grown for 3 days with 5.4 to 270 nM radioactive ^55^Fe(III)-EDTA. Cellular radioactive iron content was determined by liquid scintillation. Mean ± SD from three experiments.

Cellular iron content was determined after 3 days of incubation of exponentially growing cells in the presence of various concentrations of ^55^Fe(III)-EDTA (Fig. 3). Under iron replete condition of 270 nM total Fe, similar iron contents were detected in OTTH595 (1.1 ± 0.1 10^−15^ mol ^55^Fe cells^−1^), RCC809 (1.10 ± 0.09 10^−15^ mol ^55^Fe cells^−1^) and RCC802 (1.28 ± 0.09 10^−15^ mol ^55^Fe cells^−1^). In OTTH595 and RCC809 Fe contents remained fairly constant for Fe concentrations of 27 and 54 nM (between 0.85 ± 0.04 and 1.20 ± 0.05 10^−15^ mol ^55^Fe cells^−1^). In RCC802, in contrast, the iron content dropped progressively from 1.51 ± 0.04 down to 0.040 ± 0.003 10^−15^ mol ^55^Fe cells^−1^, corresponding to a 19 fold reduction. At 5.4 nM total Fe, RCC809 and OTTH595 also had low Fe contents (0.39 ± 0.02 and 0.22 ± 0.02 10^−15^ mol ^55^Fe cells^−1^), however no cell growth was observed at this Fe concentration for these two strains (see Fig. 1).

### Cell size and cell biomass reductions in response to iron limitation

**Figure 4:**
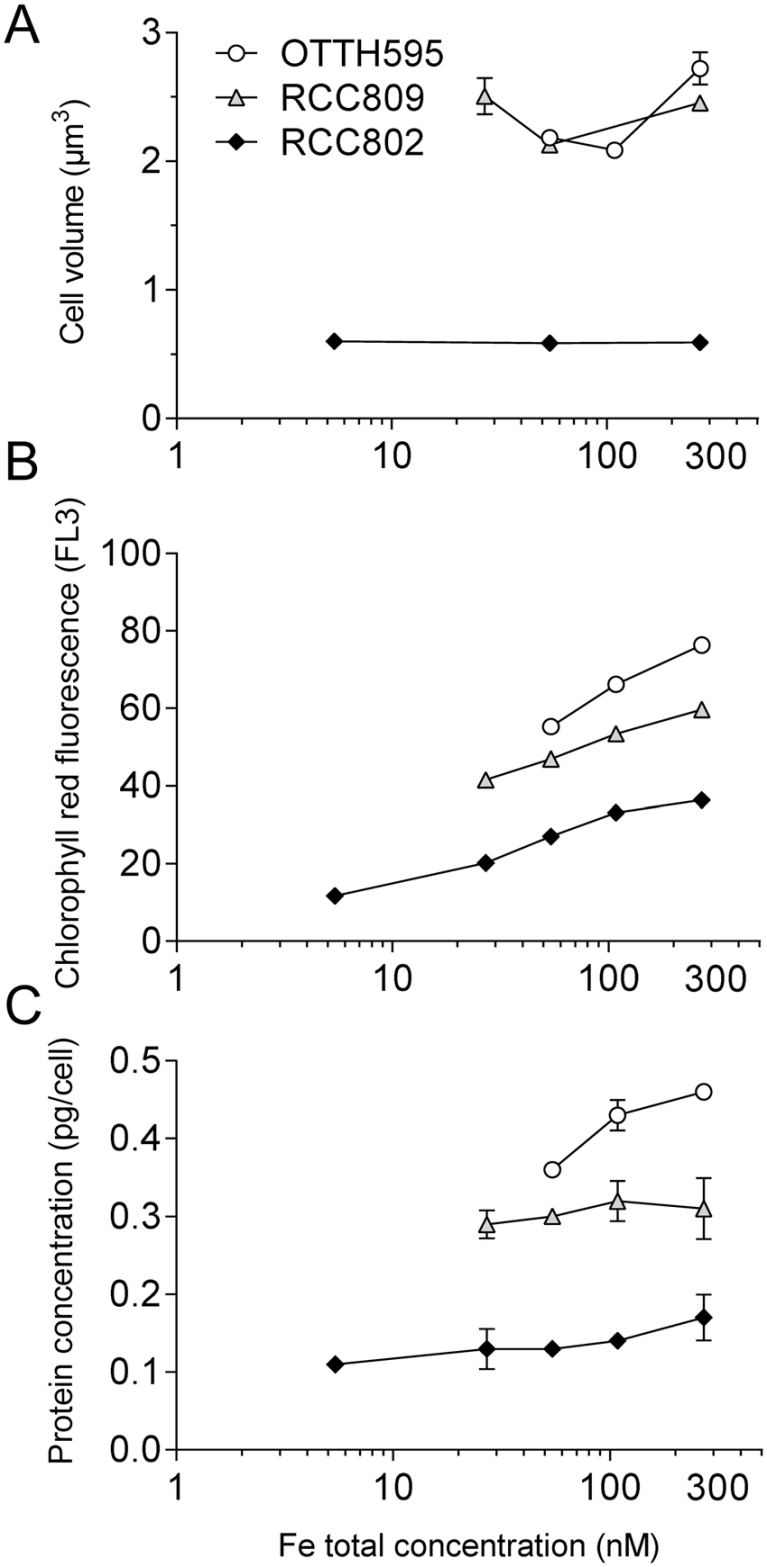
Cell biomass reduction in response to iron limitation. *Ostreococcus* strains were grown for one week in AQUIL medium containing various concentration of Fe(III)-EDTA ranging from 5.4 to 270 nM. (**A**) Cell volume inferred from cell diameter measurement. (**B**) Cellular chl fluorescence parameter measured by flow cytometry (**C**) Protein cell content. Mean ± SD from 3 experiments.

We assessed the variations of the cellular volume in response to extracellular iron concentrations (Fig. 4A). The cell surface was inferred from the average cell diameter determined using a CASY cell counter, assuming a spherical shape of these coccoid cells. When cells were transferred from iron replete conditions (270 nM total Fe) to limiting conditions, both OTTH595 and RCC809 reduced their cellular volume, from 2.72 µm^3^ to 2.09 µm^3^ under limitation (108 nM of total Fe) for OTTH595 and from 2.45 µm^3^ down to 2.12 µm^3^ at 54 nM of total Fe for RCC809. For lower iron concentrations, however, the cellular volume of RCC809 was similar to the initial value, *i*.*e*. in iron replete conditions (2.5 µm^3^). In sharp contrast, the cellular volume of the RCC802 strain was about 4 fold smaller (∼0.6 µm^3^) than the two other strains, and remained constant independently of the external iron concentrations.

The Chl red fluorescence parameter (FL3), as determined by flow cytometry, provides a reliable proxy of the Chl content in *Ostreococcus* cells (Supplementary Figure 2). Figure 4B shows that for all strains, the cell Chl fluorescence decreased in response to iron limitation from 36.4 ± 0.4 down to 11.7 ± 0.1 in RCC802), from 59.7 ± 0.3 down to 41.5 ± 0.3 in RCC809 and from 76.4 ± 0.8 down to 55.3 ± 1.3 in OTTH595. The cellular protein content was determined under the same conditions (Fig. 4C). Under iron replete conditions, the protein cell content was about 2 to 3 fold lower in RCC802 (0.170 ± 0.02 pg cell^−1^) than in OTTH595 (0.464 ± 0.005 pg cell^−1^) and RCC809 (0.3 ± 0.04 pg cell^−1^). As for the cell Chl fluorescence, the protein content of RCC802 decreased dramatically in response to iron limitation (0.11 ± 0.01 pg cell^−1^ at 5.4 nM Fe(III)-EDTA corresponding to a 35% decrease). In OTTH595 and RCC802, smaller reductions in protein contents were observed, *i*.*e*. from 0.31 down to 0.29 pg cell^−1^ in RCC809 (5% decrease) and from 0.46 to 0.36 pg cell^−1^ in OTTH595 (20% decrease).

### Normalized oxygen production of RCC802

**Figure 5:**
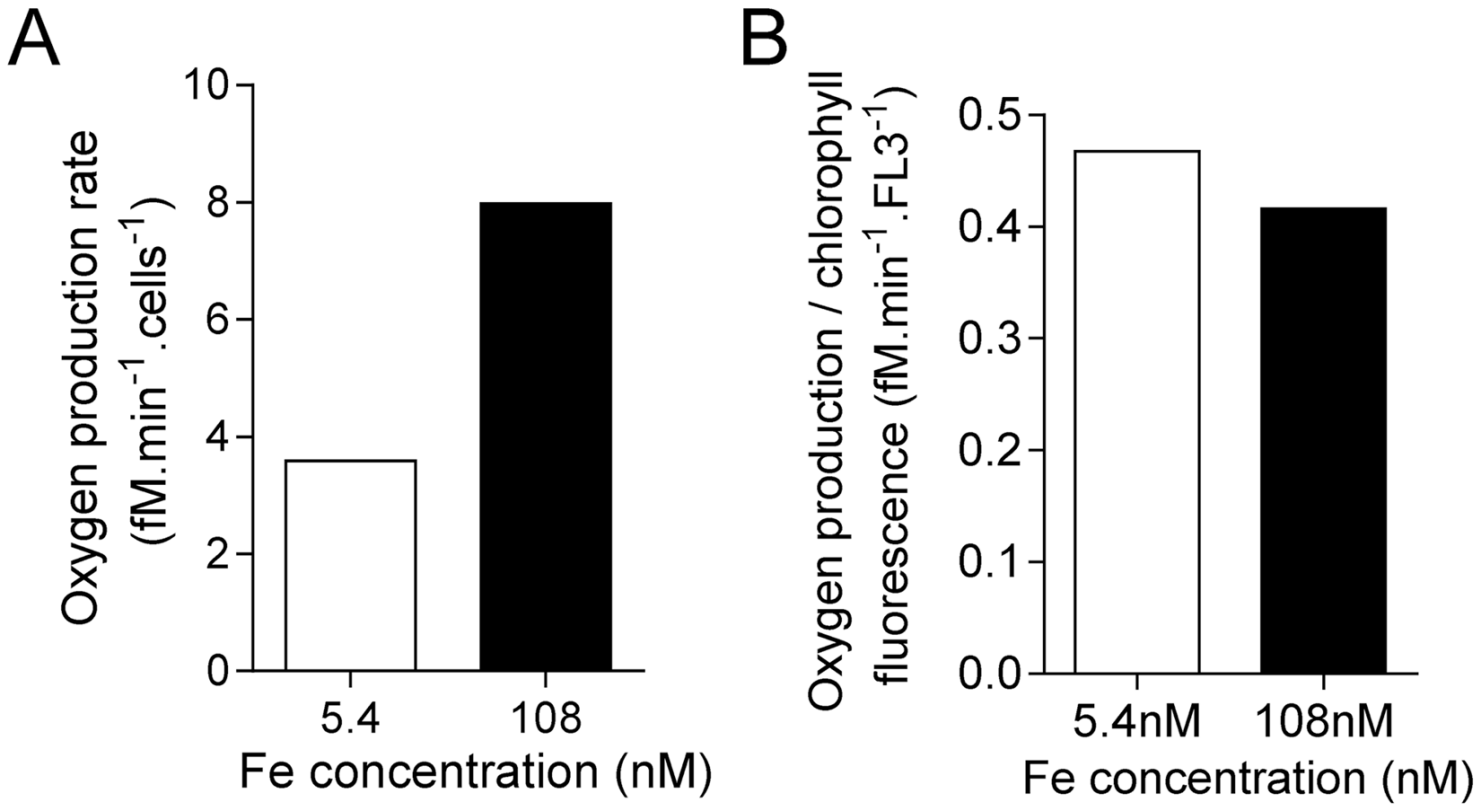
Oxygen evolving in RCC802. (**A**) Rate of net oxygen evolving in RCC802. Oxygen production resulting from photosynthesis was measured during one hour, at 20 °C under 25 µmol quanta m^−2^ s^−1^ blue light (see Supplementary Figure 2). Oxygen consumption resulting from respiration during one hour in darkness was subtracted from production to obtain the net production. (**B**) Net oxygen production determined in (**A**) normalized to the chlorophyll red fluorescence parameter (FL3) measured by flow cytometry.

The respiration and the oxygen net evolving cellular rates were measured in the RCC802 strain, under iron depleted (5.4 nM) and replete conditions (108 nM). The oxygen production, resulting from the activity of photosystems II, decreased by 50% under iron limitation, while the consumption due to respiration was four-times lower (Supplementary Figure 3). The net oxygen production (production - consumption) was reduced from 7.97 to 3.59 fM min^−1^ cell^−1^, corresponding to a 55% lowering (Fig. 5A). Interestingly, when normalized to the Chl red fluorescence, the net production of oxygen remained fairly stable in iron replete and depleted conditions (Fig. 5B).

### Transcriptomic response of RCC802 to iron limitation

**Figure 6:**
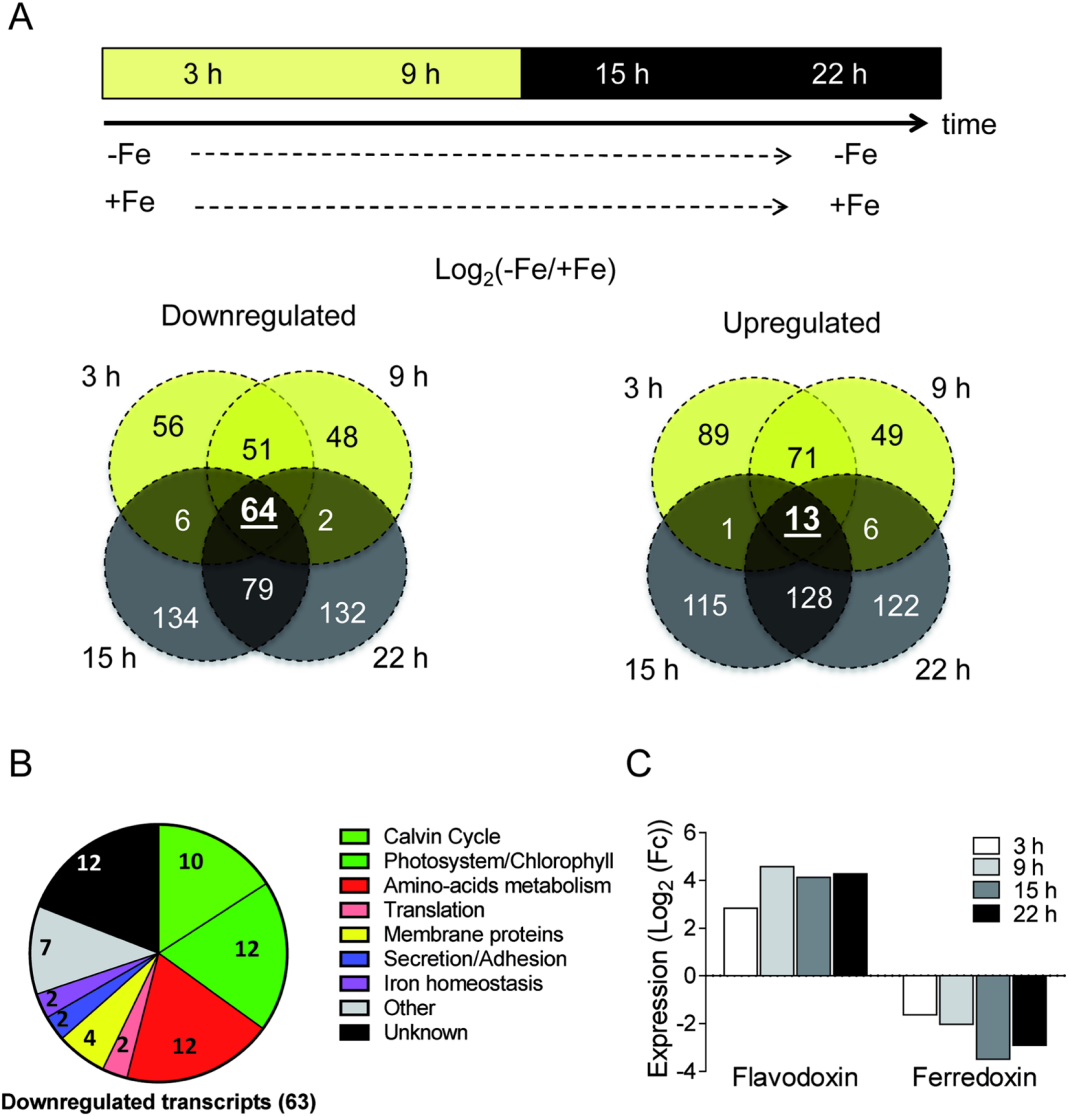
Transcriptomic response of RCC802. (**A**) RCC802 cells were grown for 5 days under 12 h light: 12 h dark cycles in +Fe (270 nM Fe(III)-EDTA) or −Fe (5.4 nM Fe(III)-EDTA) conditions. RNA samples extracted at 3 h, 9 h, 15 h and 22 h after dawn, were subjected to RNAseq analysis. Out of 16527 de novo assembled RNAs, 1251 were differentially expressed between iron depleted and replete conditions in at least one day time point (−1 < Log_2_(−Fe/+Fe) < or Log_2_(−Fe/+Fe) > 1). Venn diagrams of differentially expressed genes. Day is represented in yellow, night in dark grey. (**B**) Functional clustering of the 64 genes upregulated in iron limiting conditions. Genes encoding photosynthesis (green) and protein synthesis proteins (red) represent more than 50% of downregulated genes. (**C**) At all day times a flavodoxin was induced while the ferredoxin was repressed.

A transcriptomic study was conducted to get further insights into the regulation of mechanisms underlying acclimation and adaptation of RCC802 to iron depleted environments. RCC802 cultures were exposed to 12 h day:12 h night cycles under iron limited (5.4 nM Fe(III)-EDTA) and under iron replete conditions, for 7 days. RNA extracted from cells were harvested at 3, 9, 12, 15 and 22 h (i.e. 3 h before and after dawn and dusk) and subjected to RNAseq analysis, as described in Supplementary Figure 4. As there is no reference genome for RCC802, we undertook a *de novo* assembly approach to generate a full-length “reference transcriptome assembly” composed of all predicted transcripts produced by RCC802 in at least one experimental condition (Supplementary Figure 4). The number of reads, mapped on each reference transcript was used to calculate Log_2_(Fold Change) (LogFC) values (comparing −Fe and +Fe growth conditions) for each time point. Out of 2233 differentially expressed transcripts 980 were found to be upregulated (LogFC > 1, p-value < 0.01) and 1253 downregulated (LogFC < −1, pvalue < 0.01). After isoform filtering and removal of fusion transcripts based on systematic sequences comparisons with OTTH595, 1251 differentially expressed transcripts were kept (see Supplementary Data File 1). Based on the observation that a vast majority the genes of *O*. *tauri* are strongly regulated by the day/night cycle^43^, we focussed on genes that are induced or repressed in response to iron limitation at all day times. In iron depleted conditions 64 transcripts were downregulated (Fig. 6B, Supplementary Table 1). About one third of the sequences (23) were related to photosynthesis including (i) photosystem proteins such as chlorophyll a/b binding light-harvesting protein of PSI (Lhca proteins), and chlorophyll synthesis such as the Coproporphyrinogen III oxidase, (ii) components of the Calvin-Benson cycle such as the 1,5 ribulose bisphosphate carboxylase oxygenase (RubisCO) and the fructose-1,6-bisphosphatase. The second largest class of repressed transcripts (14) encodes enzymes involved in amino acid metabolism and components of the translation machinery such as translation elongation/initiation factors. Among the 15 remaining downregulated genes with putative homologues or functional domains in other organisms, we identified several iron binding proteins including cytochrome P450, cytochrome *b*_*561*_ and a putative mitochondrial ferric reductase (Supplementary Table 2).

Only 13 transcripts were induced across all daytimes. A RCC802 flavodoxin with no homologue in *O*. *tauri* was overexpressed in all iron-depleted conditions, whereas the iron-containing ferredoxin was repressed under the same conditions (Fig. 6C). A putative Basic Helix loop Helix transcription factor was the only transcription factor induced at all day times under iron limitation.

## Discussion

### Iron adapted strains in the genus *Ostreococcus*

Using iron limitation experiments, we were able to point out, among several *Ostreococcus* strains, differential abilities to grow in iron limiting conditions. Three different types of response are observed (Fig. 1). The coastal strains OTTH595 and RCC789 were capable of growing only under iron concentrations higher than 54 and 108 nM total Fe respectively, while the oceanic RCC809 strain showed growth down to 27 nM total Fe. RCC802, in contrast, grew well at all Fe concentrations tested with only a 20% reduction in growth rate at 5.4 nM total Fe. Comparison of growth rate inhibition between iron replete and limiting conditions clearly supports that RCC802 has the ability to grow under severe iron limitation like oceanic species such as the diatom *Thalassiosira oceanica* or the coccolithophorid *Emiliana huxlyeyi* (Table 2). In contrast, strains isolated in meso- to eutrophic environments showed much higher iron requirements. These differences in growth rate response were associated to differential physiological responses, such as the modulation of the chlorophyll and protein cell content, the photosynthetic activity, the iron cell content in response to iron limitation (Figs 1, 2, 3 and 4). RCC802 and to some extent RCC809 strains come from geographic areas where iron bioavailability is on average low, with only sporadic iron supplies from Sahara.

**Table 2:** Comparison of growth rates in different phytoplanktonic organisms under iron limiting and iron replete conditions in aquil medium (Fe(III)-EDTA source).

*Organisms*	*Growth rate ratio* −*Fe*/+*Fe*	−*Fe*/+*Fe conditions*
Pelagomonas calceolata*	0.92	4.3 nM/232 nM
Emiliana huxleyi*	0.88	3.9 nM/299 nM
Thalassiosira oceanica*	0.70	4.2 nM/303 nM
Prorocentrum minimum*	0.42	4.3 nM/299 nM
Thalassiosira weissflogii*	0.00	4.3 nM/301 nM
*Ostreococcus*‡		
RCC802	0.79	5.4 nM/270 nM
RCC809	0.14	5.4 nM/270 nM
	0.38	27 nM/270 nM
OTTH595	0.00	27 nM/270 nM
	0.40	54 nM/270 nM
RCC789	0.00	54 nM/270 nM

*From ref. 50.

^‡^This study.

Our study overall strongly supports the existence of *Ostreococcus* strains or ecotypes physiologically adapted to environments where iron concentration may be low^41^. Interestingly, RCC802 showed higher growth rates than the other strains not only under iron depleted conditions, but also following iron addition to iron-limited cells (270 nM Fe(III)-EDTA condition in Fig. 1). This suggests that this strain may not be specialized to low iron environments but rather acclimates efficiently to fluctuating environmental iron bioavailability. It is also possible that OTTH595 and RCC809 did not perform as well as RCC802 under iron replete conditions because they had been more severely limited during the acclimation phase. The cellular iron quotas of RCC802 varied by about 20 fold between iron replete and iron depleted conditions (while maintaining cellular growth rate), thereby exemplifying the plastic response of RCC802 to fluctuating iron bioavailability. OTTH595 and RCC809, in contrast, showed little variation in iron content between iron replete (270 nM) and the lowest iron concentration at which cell growth occurred (54 and 27 nM, respectively).

Field studies have revealed that *Ostreococcus* OI (clade A and C) and OII (clade B) are rarely abundant at the same location and that OI populations are dominant in coastal, cool and/mesotrophic areas wheras OII populations are more abundant in warm, deep oligotrophic regions^27^. In agreement with these data OTTH595 (clade C) exhibits higher iron requirements than the deep strain RCC809 (clade B). Surprisingly, however, RCC802 (clade A) which belongs to OI clade and was isolated at 65 m deep, has very low iron requirements compared to other strains. Together these data indicate that like light irradiance, iron is a factor which influences the ecological niche partitioning of *Ostreococcus* strains.

### Cell biomass reduction: a strategy to cope with iron limitation

Our observations showing that under iron limiting conditions, RCC802 exhibits the highest growth rate and the smallest cell diameter (compared to RCC809 and OTTH595) are consistent with the hypothesis that cell iron supply is limited by diffusion^3, 44, 45^. The smallest cells are more efficient to use iron because the supply of iron per unit of cellular volume is inversely related to the square of the cellular radius. We observed, however, that the size of RCC802 did not change when iron concentration decreased. RCC802 cells may have reach a minimal cell size, may be due to physical constraints such as mitotic spindle organization, which precludes further cell size reduction under iron limitation^46^. The main strategy of this strain to deal with iron limitation would, thus, rely on the optimisation of iron use rather than on cell size reduction. In agreement with this hypothesis we observed a nearly 20 fold decrease in intracellular iron content while maintaining cell size and growth rates under iron limitation.

Cell biomass, as estimated from the total amount of protein per cell, was much lower in RCC802 than in other strains. Compared to RCC809 and OTTH595, RCC802, showed a marked decrease of the Chl cell content, which was associated with a decrease of the oxygen net production rate per cell under iron limitation. When the oxygen net production was normalized by the Chl cell fluorescence (reflecting the Chl cell content), there was, however, no variation between iron depleted and iron replete conditions, demonstrating that the photosynthetic efficiency per Chl molecule was not affected. This is supported by the fact that the RCC802 photosystem II parameters did not significantly decrease upon iron limitation unlike in OTTH595 and RCC809, for which the growth rate decrease was associated to a pronounced drop of the photosystem II quantum yield and relative electron transport rate, strongly suggesting a global photosynthesis impairment (Fig. 2). This decrease likely originates from photosystem II photoinactivation (see e.g. ref. 47) and/or the induction of excess light energy dissipation mechanisms^23, 26^. Moreover, iron depletion induced a decrease in the light harvesting capacities in these strains and a global decrease of the Chl cell content (Figs 2C and 4B). These observations indicate that OTTH595 and RCC809 responded to iron limitation by drastically decreasing the amount of light energy utilized, which resulted in a strong decrease of the growth rate compared to RCC802 (Fig. 1). The downregulation of photosynthesis is probably the consequence of the high requirement for Fe of the photosynthetic apparatus, as characterized in other photosynthetic organisms such as diatoms^44^.

Altogether, our results suggest that, in response to iron limitation, RCC802 decreases the quantity of photosynthetic complexes per cell, *i*.*e*. the ‘volume’ of the photosynthetic machinery while maintaining its efficiency. In agreement with this hypothesis, RNAseq analysis, showed that in response to iron limitation, about one third of down regulated transcripts during all day time encode proteins involved in various aspects of photosynthesis including Chl synthesis, photosystem composition and Calvin-Benson cycle. The overall reduction in protein content is also reflected at the transcriptional level by the repression of genes involved in protein synthesis. In the low iron ecotype *Thalassiosira oceanica*, a similar reduction of the cellular protein content was probably due to a smaller number of chloroplasts per cell^44^. *Ostreococcus* species, unlike diatoms, contain a single chloroplast per cell, which occupies about 50% of the cell volume. The reduction of protein and chlorophyll content per cell in RCC802 is therefore likely to reflect a global reduction of the photosynthetic machinery and of the chloroplast. When comparing the expression patterns of the 64 RCC802 downregulated transcripts with putative homologues in OTTH595, we found that most genes were not downregulated in this latter species (Supplementary Table 3). This observation is consistent with the fact that OTTH595 does not reduce as much its cellular biomass under iron limitation.

Adaptations relying on the regulation of iron binding proteins are also suggested by the transcriptomic analyses. For example, an RCC802 flavodoxin, which had no homologue in OTTH595 or RCC809, is upregulated under iron limitation, while ferredoxin is downregulated (Fig. 6) suggesting that in RCC802 like in oceanic diatoms, the substitution of ferredoxin by flavodoxin may contribute to the ecological success under chronically low iron environments^5–7^. The single *BHLH* gene is the only transcription factor of RCC802 to be constitutively induced under iron limitation. BHLH, in contrast, was down regulated in OTTH595 under iron limitation. BHLH may, therefore, be a transcriptional regulator involved in the efficient acclimation of RCC802 to low iron environments, like the*BHLH Fer* gene of higher plants^48^.

## Conclusions

Our results establish the existence of an *Ostreococcus* “low iron requiring strain”, which acclimates efficiently to low iron conditions. They support the field studies suggesting that picoeukaryotes should not be seen only as components of mesotrophic areas^27^ and suggest that iron may drive the differentiation of physiologically specialized strains along coast-ocean gradients. The main acclimation to low iron environment by *Ostreococcus* sp. RCC802 appears to involve primarily a reduction of cell biomass, rather than the reduction of cell surface/volume ratio reported in nano and microphytoplankton.

## Electronic supplementary material

Supplementary information

Supplementary information
