# Supplementary material for: Acclimation of a low iron adapted Ostreococcus strain to iron limitation through cell biomass lowering
Source: Sci Rep. 2017 Mar 23;7:327. doi: 10.1038/s41598-017-00216-6 (PMC5428002; doi:10.1038/s41598-017-00216-6)
Supplement: Supplementary file 1 — Supplementary information [file 41598_2017_216_MOESM1_ESM.pdf]

SUPPLEMENTARY INFORMATION

Acclimation of a low iron adapted *Ostreococcus* strain to iron limitation through cell biomass lowering

Hugo Botebol, Gaelle Lelandais, Christophe Six, Emmanuel Lesuisse, Arnaud Meng, Lucie Bittner, Stéphane Lecrom, Robert Sutak, Jean-Claude Lozano, Philippe Schatt, Valérie Vergé, Stéphane Blain and François-Yves Bouget

SUPPLEMENTARY INFORMATION

Supplementary Materials and Methods

Measure of light response using Pulse amplitude modulated fluorometry

After 5 min relaxation in darkness, the non-actinic modulated light (450 nm) was turned on in order to measure the fluorescence basal level, F_0. A saturating red light pulse (655 nm, 4 000 µmol quanta m^{-2} s^{-1}, 400 ms) was applied to determine the maximum fluorescence level in the dark adapted sample, F_M. The maximal PSII fluorescence quantum yied of photochemical energy conversion, F_V/F_M, was calculated using the following formula:

$$F_V/F_M = (F_M – F_0) / F_M$$

The sample was then submitted to a series of 11 red light irradiance (655 nm) steps (of 50 s each) increasing from dark to ca. 2 000 µmol quanta m^{-2} s^{-1} and a single saturating light pulse was triggered after each step. The maximal fluorescence levels in light adapted samples, F_M', were then determined and the effective quantum yield was calculated according to Genty *et al.* (1989), as follows:

$$\phi_{PSII} = (F_M' – F_t) / F_M'$$

where F_t is the fluorescence steady state level immediately prior to the flash. The relative electron transport rate (rETR) was estimated according the modified formula of Genty *et al.*, (1989) :

$$rETR = \phi_{PSII} \times I$$

where I is the irradiance in µmol quanta m^{-2} s^{-1}. The photosynthetic parameters, the initial slope of the rETR *vs.* I curve, the maximal rETR (rETRmax) and the subsaturating irradiance (I_K) were determined by fitting the photosynthesis model for phytoplankton from Eilers & Peeters, (1988). Measurements were made on cell cultures in late exponential phase.

Determination of chlorophyll content

For Chlorophyll content determination, a volume of about 15 ml of culture in exponential phase was centrifuged at 10.000 *g* (10 min, 4°C). The pellet was resuspended in 180 µl 100% cold methanol, stored at -20°C for 30 min prior to centrifugation at 20.000 *g* to remove all particles and cell debris. The supernatant was then brought to 10% Milli-Q water to avoid peak distortion and a volume of 100 µl of the pigment extract was immediately injected into

an HPLC Hewlett-Packard HPLC 1100 Series system, equipped with a quaternary pump and diode array detector. Pigment separation was performed using a Waters Symmetry C_8 column (150 x 3 x 4.6 mm, 3.5 µm particle size) according to procedures published elsewhere (Zapata *et al.*, 2000; Six *et al.*, 2005) at a flow rate of 1 ml min^{-1}. Chlorophylls were detected by their absorbance at 440 nm and identified by diode array spectroscopy.

Supplementary Table 1

TRINITY transcript	Function	Putative protein/domain	Length (nt)	Best Blast hits	
				O. tauri	*O. lucimarinus*
comp713_c0_seq1	**PHOTOSYNTHESIS**	triose phosphate transporter(calvin cycle)	1245	ostta05g01630	Ol05g01680
comp2766_c0_seq1		Phosphoglycerate kinase (calvin cycle)	1450	ostta06g00700	Ol06g00640
comp2150_c0_seq1		Glyceraldehyde 3-phosphate dehydrogenas	1209	ostta01g01560	Ol01g01520
comp2891_c0_seq1		protein FLUORESCENT IN BLUE LIGHT chloroplast	586	ostta06g04520	Ol06g04510
comp2779_c0_seq1		Glyceraldehyde 3-phosphate dehydrogenase,	244	ostta01g01560	Ol10g01510
comp721_c0_seq1		transketolase chloroplast precursor	2333	ostta07g04370	Ol07g04340
comp1607_c0_seq1		Ribulose bisphosphate carboxylase small chain	655	NA	Ol17g01940
comp2027_c0_seq1		Ferredoxin [2Fe-2S], plant	557	ostta17g00310	Ol16g00410
comp2898_c0_seq1		Chlorophyll a/b binding protein domain	294	ostta02g03330	Ol02g01460
comp2899_c0_seq1		Chlorophyll a/b binding protein domain	891	ostta05g03830	Ol05g03870
comp867_c0_seq1		Photosystem II PsbQ, oxygen evolving complex	805	ostta16g01620	Ol15g01250
comp765_c0_seq1		chlorophyll a/b binding light-harvesting protein of photosystem I	1013	ostta14g00065	Ol20g01170
comp2834_c0_seq1		Photosystem I PsaN	513	ostta06g00250	Ol06g00210
comp753_c0_seq1		chlorophyll a/b binding light-harvesting protein of photosystem I	931	ostta03g04920	Ol03g04970
comp3131_c0_seq1		Coproporphyrinogen III oxidase (chlorophyll synthesis)	1490	ostta03g03160	Ol03g03180
comp2244_c0_seq1		terpenoid synthase (chlorophyll/carotenoid precusors)	1064	ostta12g01280	Ol12g01300
comp2922_c0_seq1		Chlorophyll a/b binding protein	805	ostta02g04800	Ol02g04300
comp2437_c0_seq1		psaO, PSI-O, photosystem I	501	ostta02g05340	Ol02g04990

comp1546_c0_seq2		chlorophyll a/b binding,photosystem I light harvesting complex	806	ostta09g04420	Ol02g01460
comp2066_c0_seq1		glyceraldehyde-3-phosphate dehydrogenase subunit A	1288	ostta10g03420	Ol10g03460
comp2774_c0_seq1		Glyceraldehyde 3-phosphate dehydrogenase	337	ostta01g01560	Ol01g01510
comp2775_c0_seq1		ribulose bisphosphate carboxylase/oxygenase activase	1394	ostta04g02510	Ol04g02600
comp2939_c0_seq1		Fructose-1,6-bisphosphatase	1265	ostta03g00350	Ol03g00290
comp2481_c1_seq1	**AMINO-ACID**	asparagine synthase	991	ostta01g03620	Ol01g03890
comp740_c0_seq1	**METABOLISM**	2-oxoglutarate/malate translocator	2630	ostta18g00620	Ol17g00650
comp4887_c0_seq1		Glycine cleavage H protein	350	ostta15g02900	NA
comp2790_c0_seq1		Serine hydroxymethyltransferase	1674	ostta14g02420	Ol20g02600
comp708_c0_seq1		S-adenosyl-L-homocysteine hydrolase	1734	ostta09g02610	Ol09g02440
comp686_c0_seq1		Glycine cleavage H-protein	657	ostta15g02900	Ol14g03890
comp709_c0_seq1		Pyridoxal phosphate-dependent transferase	1493	ostta18g01090	Ol17g01100
comp1732_c0_seq1		AAP amino acid transporter	1448	ostta16g02660	Ol15g02530
comp2100_c0_seq5		Aspartate semi-aldehyde dehydrogenesase (Methionin)	1258	ostta01g02480	Ol01g02500
comp2692_c1_seq1		HAD hydrolase	1190	ostta06g04120	Ol15g02100
comp1553_c0_seq1		Pyridoxal phosphate-dependent transferase	3050	ostta10g03200	Ol10g03180
comp703_c0_seq1		Glycine cleavage system T protein	1316	ostta10g02780	Ol10g02750
comp3393_c0_seq1	**TRANSLATION**	Triger factor	2119	ostta02g02090	Ol02g04150
comp2792_c0_seq1		Translation elongation/initiation factor	2343	ostta07g04530	Ol07g04490
comp807_c0_seq2	**IRON HOMEOSTASIS**	Ferric reductase transmembrane domain (mitochondrial)	2732	ostta09g01890	Ol16g00340
comp810_c0_seq2		Cytochrome b561/ferric reductase (heme binding)	517	ostta04g02480	Ol17g00350
comp3928_c0_seq1	**MEMBRANE**	tetratricopeptide repeat	1036	ostta17g01220	Ol16g01350

comp1076_c0_seq2		tetratrico peptide repeat	1242	ostta15g02730	Ol14g03690
comp3743_c0_seq1		ABC transporter	735	ostta09g03190	Ol09g02910
comp715_c0_seq1		putative transmembrane protein	1019	ostta01g04420	Ol01g04590
comp2817_c0_seq1	**SECRETION, ADHESION**	GRASP55/65	780	ostta04g00130	Ol04g00120
comp1598_c0_seq1		fasciclin	731	ostta13g00230	Ol13g00190
comp2945_c0_seq1	**STRESS RESPONSE**	glutathione peroxidase	1079	ostta08g03450	Ol08g03380
comp3713_c0_seq1		Cytochrome P450, E-class (heme binding)	2238	ostta07g00380	Ol07g00360
comp2785_c0_seq1	**RNA PROCESSING**	fibrillarin	646	ostta07g01205	Ol07g01220
comp2861_c0_seq1	**FATTY ACID SYNTHESIS**	acyl carrier protein	529	ostta18g01380	Ol12g01140
comp710_c0_seq1	**CHROMATIN STRUCTURE**	SNF2-related	1648	ostta09g01150	Ol07g00940
comp1737_c0_seq1	**CIRCADIAN CLOCK**	Constans	1474	ostta09g01510	Ol09g01380
comp1610_c0_seq1		Rhodanese like domain (sulfur transferase)	802	NA	Ol05g04620
comp3394_c0_seq1	**UNKNOWN**	unknown	375	NA	Ol08g01030
comp662_c0_seq1		unknown	1823	ostta14g02460	Ol20g02650
comp2990_c0_seq1		unknown	848	NA	NA
comp712_c0_seq2		unknown	489	ostta09g01010	Ol17g01650
comp2789_c0_seq1		unknown	369	NA	Ol10g00430
comp2806_c0_seq1		unknown	980	ostta01g05100	Ol03g00540
comp2897_c0_seq1		unknown	555	ostta12g00390	Ol12g00490
comp2441_c1_seq2		unknown	1102	ostta08g03740	Ol08g03680
comp1958_c0_seq1		unknown	428	NA	NA
comp2761_c0_seq1		unknown	535	ostta11g01390	Ol11g01480

comp1875_c0_seq1	unknown	606	NA	NA
comp2784_c0_seq1	unknown	787	NA	Ol04g01670

Supplementary Table 1: . List of the 63 RCC802 putative transcripts that are down-regulated in iron depleted conditions. BlastX were performed on *O. tauri* and *O. lucimarinus* predicted proteins at http://bioinformatics.psb.ugent.be/orcae/. NA means that no putative homologue was detected.

Supplementary Table 2

TRINITY transcript	Function	Putative protein/domain	Length (nt)		
				O. tauri	O. lucimarinus
comp1191_c0_seq1	TRANSCRIPTION	Myc-type, basic helix-loop-helix (bHLH) domain	2270	ostta14g01990	Ol20g02150
comp2507_c0_seq2	PHOTOSYNTHESIS	flavodoxin (FMN dependent)	2032	NA	Ol08g01470
comp1591_c0_seq2	PHOTOSYNTHESIS	ribulose phosphate 3 epimerase	1630	ostta06g01650	Ol06g01590
comp3218_c0_seq1	PHOTOPROTECTION	Violaxanthin de-epoxidase related	1554	ostta09g01160	Ol07g00920
comp1792_c0_seq1	PHOTORESPIRATION	glycolate oxidase (Alpha-hydroxy acid dehydrogenase, FMN-dependent)	4433	ostta17g02350	Ol14g02530
comp74_c0_seq1	RESPIRATION	2-oxoglutarate/malate carrier protein (glyoxylate cycle shunt)	1312	ostta09g00260	Ol12g01810
comp3155_c0_seq1	PROTEIN FOLDING	HSP20-like chaperone	1549	ostta03g02290	Ol03g02330
comp2824_c0_seq1	DNA REPLICATION	Ribonucleoside-diphosphate reductase small chain (Fe cofactor, ferritin superfamily),	1279	ostta08g00560	Ol08g00490
comp2268_c0_seq1	SECRETION	Clathrin adaptor	2072	ostta13g01010	Ol13g00960
comp5052_c0_seq1	MEMBRANE	Ankyrin repeat-containing domain	1381	NA	Ol20g02400
comp1527_c0_seq2	OTHER	viral reverse transcriptase	1606	NA	Ol18g00290
comp1584_c0_seq1	MEMBRANE	Ankryn repeat protein	608	NA	Ol07g04470
comp3752_c0_seq1	UNKNOWN	Unknown	1455	ostta10g03510	Ol10g03610

Supplementary Table 2: List of the 13 RCC802 putative transcripts that are up-regulated in iron depleted conditions. BlastX were performed on O. tauri and O. lucimarinus predicted proteins at http://bioinformatics.psb.ugent.be/orcae/. NA means that no putative homologue was detected.

Dowregulated transcripts in RCC802

TRINITY transcript	Function	Length (nt)	Best Blast hits	RNAseq OTTH695 (Lelandais et al., BMC Genomics, 2016)				RNAseq RCC802 (this study)			
			O. tauri	logFC T1	logFC T2	logFC T3	logFC T4	logFC T1	logFC T2	logFC T3	logFC T4
comp713_c0_seq1	**PHOTOSYNTHESIS**	1245	ostta05g01630	-0,55	0,57	0,35	-0,45	-2,72	-2,88	-2,22	-1,95
comp2766_c0_seq1		1450	ostta06g00700	*-1,69*	*-0,39*	*0,43*	*-0,51*	-2,60	-3,47	-3,71	-3,25
comp2150_c0_seq1		1209	ostta01g01560	-1,05	-0,39	0,73	-0,06	-2,22	-2,38	-2,29	-1,27
comp2891_c0_seq1		586	ostta06g04520	-0,33	-0,14	1,48	-0,04	-2,09	-2,25	-2,66	-2,44
comp2779_c0_seq1		244	ostta01g01560	-1,05	-0,39	0,73	-0,06	-1,93	-2,61	-3,11	-2,46
comp721_c0_seq1		2333	ostta07g04370	*-1,28*	*0,00*	*-0,27*	*-1,05*	-1,65	-2,02	-3,10	-3,14
comp1607_c0_seq1		655	NA					-1,65	-2,35	-2,72	-2,90
comp2027_c0_seq1		557	ostta17g00310	*-0,89*	*0,37*	*0,44*	*-0,33*	-1,63	-2,02	-3,49	-2,90
comp2898_c0_seq1		294	ostta02g03330	*-0,06*	*-0,17*	*1,94*	*1,05*	-1,57	-1,40	-1,43	-1,47
comp2899_c0_seq1		891	ostta05g03830	*-0,24*	*-0,58*	*2,85*	*2,23*	-1,57	-1,69	-2,24	-2,09
comp867_c0_seq1		805	ostta16g01620	*-0,41*	*-0,35*	*1,15*	*1,08*	-1,42	-1,61	-2,36	-2,57
comp765_c0_seq1		1013	ostta14g00065	*-0,05*	*-0,02*	*3,16*	*0,61*	-1,30	-1,15	-1,92	-2,06
comp2834_c0_seq1		513	ostta06g00250	*-0,07*	*-0,15*	*0,70*	*0,55*	-1,24	-1,26	-1,93	-1,22
comp753_c0_seq1		931	ostta03g04920	*-0,11*	*-0,24*	*2,26*	*1,64*	-1,23	-1,16	-1,33	-1,41
comp3131_c0_seq1		1490	ostta03g03160	*-0,80*	*1,13*	*1,89*	*-0,21*	-1,19	-1,05	-1,91	-2,53
comp2244_c0_seq1		1064	ostta12g01280	*-0,57*	*-0,03*	*0,54*	*-0,49*	-1,18	-1,44	-1,05	-1,81
comp2922_c0_seq1		805	ostta02g04800	*-0,03*	*-0,10*	*1,58*	*1,22*	-1,12	-1,01	-1,73	-1,35
comp2437_c0_seq1		501	ostta02g05340	*-0,10*	*-0,10*	*0,74*	*0,99*	-1,02	-1,11	-1,49	-1,58

comp1546_c0_seq2		806	ostta09g04420	-0,20	-0,12	1,95	1,19	-1,10	-1,08	-1,96	-1,37
comp2066_c0_seq1		1288	ostta10g03420	-1,14	-0,90	0,00	-0,26	-1,98	-2,36	-2,73	-2,64
comp2774_c0_seq1		337	ostta01g01560	-1,05	-0,39	0,73	-0,06	-1,70	-2,50	-3,16	-2,51
comp2775_c0_seq1		1394	ostta04g02510	-0,99	0,09	0,66	-0,49	-1,60	-2,12	-2,29	-1,78
comp2939_c0_seq1		1265	ostta03g00350	-0,54	0,63	1,95	-0,21	-1,73	-1,77	-2,69	-3,08
comp2481_c1_seq1	**AMINO-ACID**	991	ostta01g03620	-0,34	-0,37	-0,31	-0,50	-2,04	-2,28	-2,21	-1,55
comp740_c0_seq1	**METABOLISM**	2630	ostta18g00620	-0,79	0,04	-0,19	0,13	-2,01	-2,22	-2,29	-1,60
comp4887_c0_seq1		350	ostta15g02900	-0,85	0,10	0,59	-0,23	-1,95	-1,90	-3,88	-1,74
comp2790_c0_seq1		1674	ostta14g02420	-1,29	-0,13	0,31	-0,44	-1,47	-1,58	-1,51	-1,55
comp708_c0_seq1		1734	ostta09g02610	-0,24	1,34	0,68	-1,29	-1,38	-1,31	-1,26	-1,30
comp686_c0_seq1		657	ostta15g02900	-0,85	0,10	0,59	-0,23	-1,30	-1,64	-1,16	-1,50
comp709_c0_seq1		1493	ostta18g01090	-1,21	-0,37	0,11	0,02	-1,14	-1,39	-1,41	-1,34
comp1732_c0_seq1		1448	ostta16g02660	-0,41	0,07	0,13	0,32	-1,01	-1,04	-1,09	-1,07
comp2100_c0_seq5		1258	ostta01g02480	-0,18	0,16	0,01	-0,04	-1,01	-1,16	-1,26	-1,09
comp2692_c1_seq1		1190	ostta06g04120	-0,93	-0,18	0,45	0,42	-1,04	-1,21	-1,02	-1,02
comp1553_c0_seq1		3050	ostta10g03200	-1,22	-0,10	0,51	-0,27	-1,70	-1,84	-1,24	-1,89
comp703_c0_seq1		1316	ostta10g02780	-1,05	0,14	0,64	0,13	-1,75	-2,05	-1,72	-1,11
comp3393_c0_seq1	**TRANSLATION**	2119	ostta02g02090	-0,81	0,32	-0,72	-0,68	-1,38	-2,35	-3,13	-3,07
comp2792_c0_seq1		2343	ostta07g04530	-1,03	1,57	-0,22	-0,98	-1,09	-1,12	-1,88	-2,85
comp807_c0_seq2	**IRON**	2732	ostta09g01890	-0,85	-1,00	-0,44	-0,53	-1,69	-1,70	-2,38	-1,65
comp810_c0_seq2	**HOMEOSTASIS**	517	ostta04g02480	-0,35	0,01	-0,70	-0,66	-1,50	-1,65	-2,50	-1,22
comp3928_c0_seq1	**MEMBRANE**	1036	ostta17g01220	-0,42	0,17	-0,08	-0,34	-1,63	-2,17	-2,37	-1,96

comp1076_c0_seq2		1242	ostta15g02730	-0,17	-0,34	-0,60	-0,34	-1,87	-2,19	-1,90	-1,37
comp3743_c0_seq1		735	ostta09g03190	-0,47	-0,21	-0,46	-0,25	-1,18	-1,54	-1,51	-1,37
comp715_c0_seq1		1019	ostta01g04420	-1,39	-1,27	-0,82	-1,11	-1,79	-2,47	-1,89	-2,27
comp2817_c0_seq1	**SECRETION**	780	ostta04g00130	-0,88	-0,18	0,28	0,23	-1,79	-2,09	-2,46	-1,48
comp1598_c0_seq1	**ADHESION**	731	ostta13g00230	-1,47	-0,39	0,56	-0,59	-2,45	-3,42	-3,74	-2,56
comp2945_c0_seq1	**STRESS RESPONSE**	1079	ostta08g03450	-1,55	-0,35	-0,86	-1,35	-2,37	-3,49	-3,90	-3,90
comp3713_c0_seq1		2238	ostta07g00380	-0,70	-0,19	0,15	-0,44	-1,53	-1,24	-1,83	-2,55
comp2785_c0_seq1	**RNA PROCESSING**	646	ostta07g01205	-0,63	0,55	1,27	-0,01	-1,78	-2,26	-2,76	-3,18
comp2861_c0_seq1	**FATTY ACID**	529	ostta18g01380	0,14	0,41	0,42	0,63	-1,02	-1,03	-1,50	-1,38
comp710_c0_seq1	**CHROMATIN**	1648	ostta09g01150	1,27	-0,19	-1,35	-2,27	-1,24	-1,74	-2,04	-2,09
comp1737_c0_seq1	**CIRCADIAN CLOCK**	1474	ostta09g01510	-0,72	-1,07	-0,82	-0,20	-1,69	-1,68	-1,52	-1,36
comp1610_c0_seq1		802	NA					-3,02	-3,68	-4,08	-3,21
comp3394_c0_seq1	**UNKNOWN**	375	NA					-2,51	-2,64	-4,22	-2,57
comp662_c0_seq1		1823	ostta14g02460	3,87	-0,17	-0,28	-0,43	-2,27	-2,36	-2,46	-1,85
comp2990_c0_seq1		848	NA					-1,66	-1,47	-1,72	-1,86
comp712_c0_seq2		489	ostta09g01010	-1,21	-0,37	0,49	-0,23	-1,62	-2,22	-3,12	-2,23
comp2789_c0_seq1		369	NA					-1,59	-1,83	-2,64	-1,78
comp2806_c0_seq1		980	ostta01g05100	0,52	2,00	-0,66	-1,08	-1,49	-1,40	-1,05	-1,19
comp2897_c0_seq1		555	ostta12g00390	-0,49	0,15	1,42	0,95	-1,45	-1,33	-1,49	-1,73
comp2441_c1_seq2		1102	ostta08g03740	-0,61	-0,22	-0,16	-0,53	-1,41	-1,75	-1,51	-1,25
comp1958_c0_seq1		428	NA					-1,27	-1,27	-1,33	-1,75
comp2761_c0_seq1		535	ostta11g01390	-0,38	-0,80	-0,01	-0,44	-1,27	-2,02	-1,50	-1,40

comp1875_c0_seq1	606	NA	-1,19	-1,12	-1,80	-1,43
comp2784_c0_seq1	787	NA	-2,57	-3,08	-4,30	-3,49

Upregulated transcripts in RCC802

TRINITY transcript	Putative protein/domain	Length (nt)		RNAseq OTTH595 (Lelandais et al., BMC Genomics, 2016)				RNAseq RCC802 (this study)			
			O. tauri	logFC T1	logFC T2	logFC T3	logFC T4	logFC T1	logFC T2	logFC T3	logFC T4
comp1191_c0_seq1	Myc-type, basic helix-loop-helix (bHLH) domain	2270	ostta14g01990	-0,51	-0,34	-0,12	-0,59	2,00	2,85	3,14	3,26
comp2507_c0_seq2	flavodoxin (FMN dependent)	2032	NA					2,84	4,58	4,13	4,27
comp1591_c0_seq2	ribulose phosphate 3 epimerase	1630	ostta06g01650	-0,42	1,64	1,19	-0,35	2,20	5,08	6,21	inf
comp3218_c0_seq1	Violaxanthin de-epoxidase related	1554	ostta09g01160	0,78	2,02	0,73	2,24	2,20	5,08	6,21	Inf
comp1792_c0_seq1	glycolate oxidase (Alpha-hydroxy acid dehydrogenase, FMN-dependent)	4433	ostta17g02350	0,40	-0,40	0,58	-0,04	2,20	5,08	6,21	Inf
comp74_c0_seq1	2-oxoglutarate/malate carrier protein (glyoxylate cycle shunt)	1312	ostta09g00260	-0,06	-0,34	0,96	1,31	1,93	2,54	4,56	1,86
comp3155_c0_seq1	HSP20-like chaperone	1549	ostta03g02290	0,78	0,79	0,85	0,59	1,71	2,13	1,71	1,07
comp2824_c0_seq1	Ribonucleoside-diphosphate reductase small chain (Fe cofactor, ferritin superfamily),	1279	ostta08g00560	2,21	0,20	0,35	0,54	1,78	2,14	3,06	2,51
comp2268_c0_seq1	Clathrin adaptor	2072	ostta13g01010	0,13	-0,14	0,07	1,07	1,10	1,21	1,17	1,79
comp5052_c0_seq1	Ankyrin repeat-containing domain	1381	NA					1,73	4,12	2,62	1,31
comp1527_c0_seq2	viral reverse transcriptase	1606	NA					1,36	2,29	2,02	1,25

comp1584_c0_seq1	Ankryn repeat protein	608	NA					1,27	1,29	3,10	2,89
comp3752_c0_seq1	Unknown	1455	ostta10g03510	-0,38	-0,12	0,41	-0,19	1,23	1,81	1,63	1,86

Supplementary Table 3: .Comparison of expression patterns of RCC802 transcripts (listed in Table S2) and putative *O. tauri* homologues in iron depleted conditions (data from Lelandais et al., 2016). NA means that no putative homologue was detected. Downregulated RCC802 transcripts under all conditions with homologues in OTTH595(Top of the table), Upregulated RCC802 transcripts under all conditions with homologues in OTTH595 (Bottom of the table). Upredgulated genes are represented in red, downregulated in green. Inf : the reference was to low, to calculate a logFC ratio. RCC802 and OTTH595 cultures were grown in parallel and harvested at the same time points T1 to T4 (corresponding to time 3h, 15h, 19h and 22h under 12:12 day/night conditions) .

Supplementary Figures

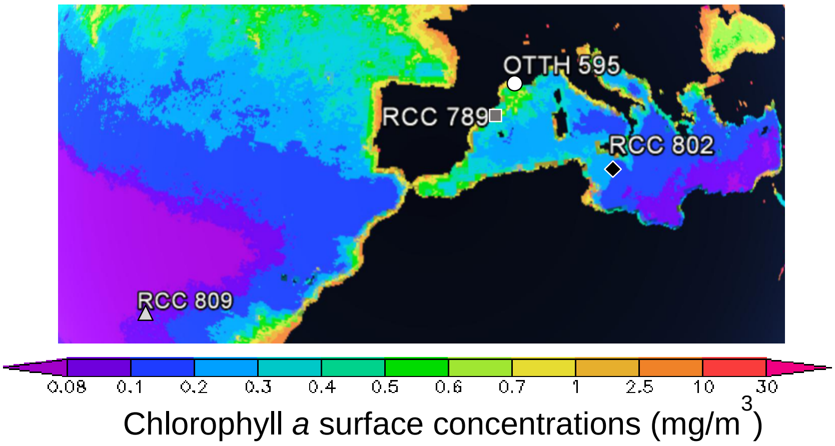

Supplementary Figure 1: Map showing the average surface concentration of Chl *a*, reflecting the seawater trophic regimes (MODIS-Aqua 4 km data collected from December 2012 to January 2013, produced with the Giovanni online data system, NASA GES DISC; https://disc.gsfc.nasa.gov/gesNews/giovanni_3_end_of_service?instance_id=ocean_month). Isolation sites are shown for OTTH595 (open circles), RCC789 (grey squares), RCC809 (grey triangles) and RCC802 (black diamond) *Ostreococcus* strains.

A

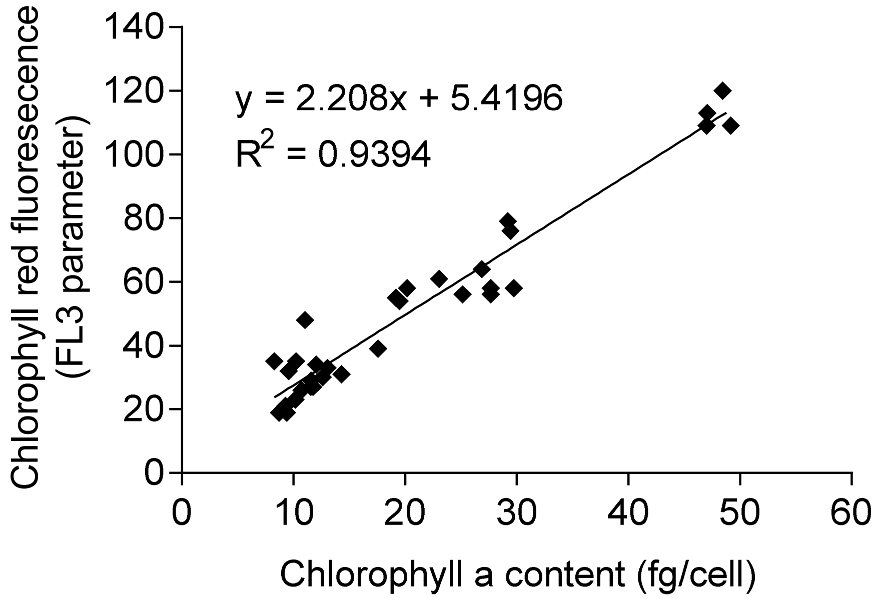

B

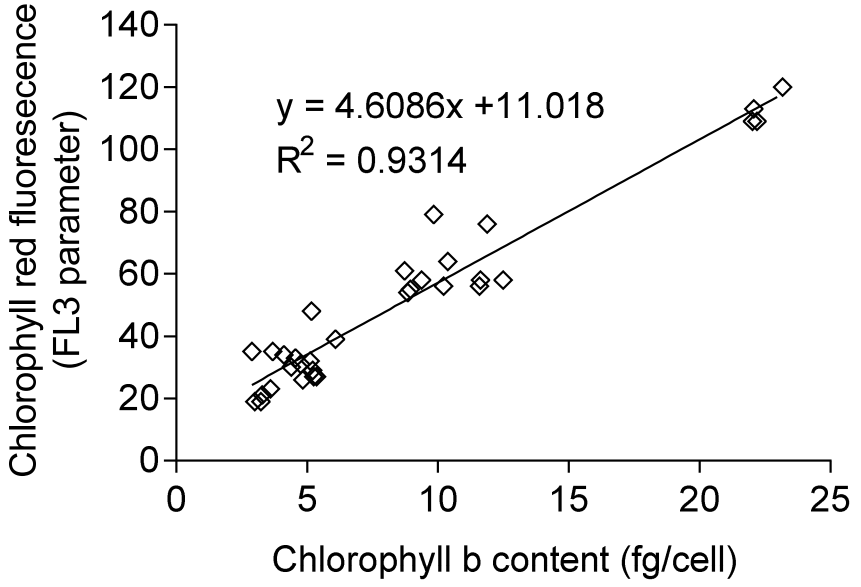

Supplementary Figure 2: Correlation between chlorophyll a **(A)** and chlorophyll b **(B)** contents as determined by HPLC quantitation and the flow cytometry red fluorescence parameter (FL3). The data plotted were obtained from cell cultures grown at various light intensities. These graphs illustrate that the FL3 value provides a good proxy of Chlorophyll a and b relative content.

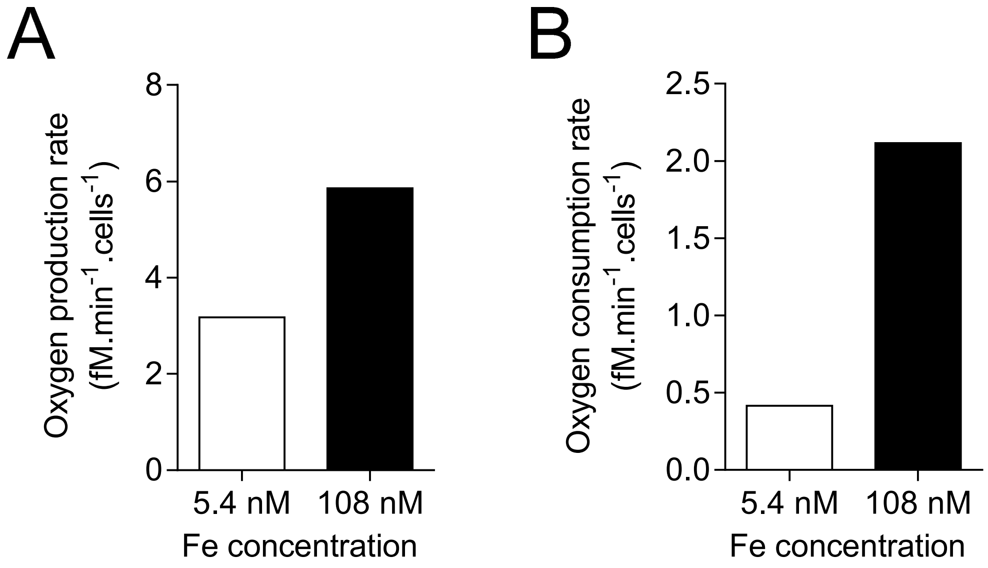

Supplementary Figure 3: Rates of oxygen production and consumption. RCC802 cells were grown for 1week in iron limited and iron replete conditions, corresponding to. 5.4 nM and 108 nM Fe(III)-EDTA**. A.** Oxygen production rates measured during 1h under blue light irradiance at 20 µmole.quanta.m^{-2}.s^{-1}**. B.** Oxygen consumption rates were subsequently monitored during 1h in the dark.

Supplementary Figure 4

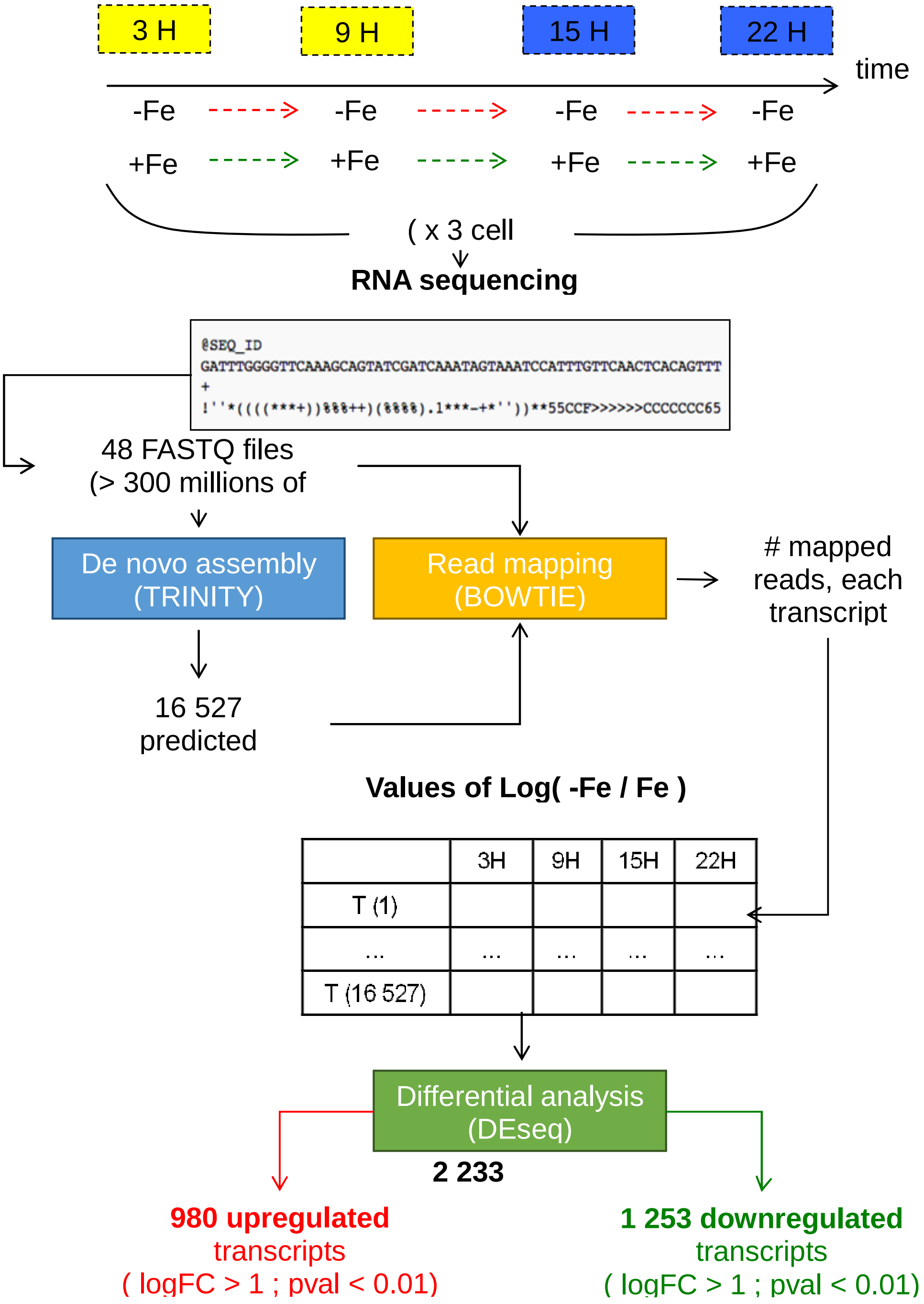

Figure S4 (continued)

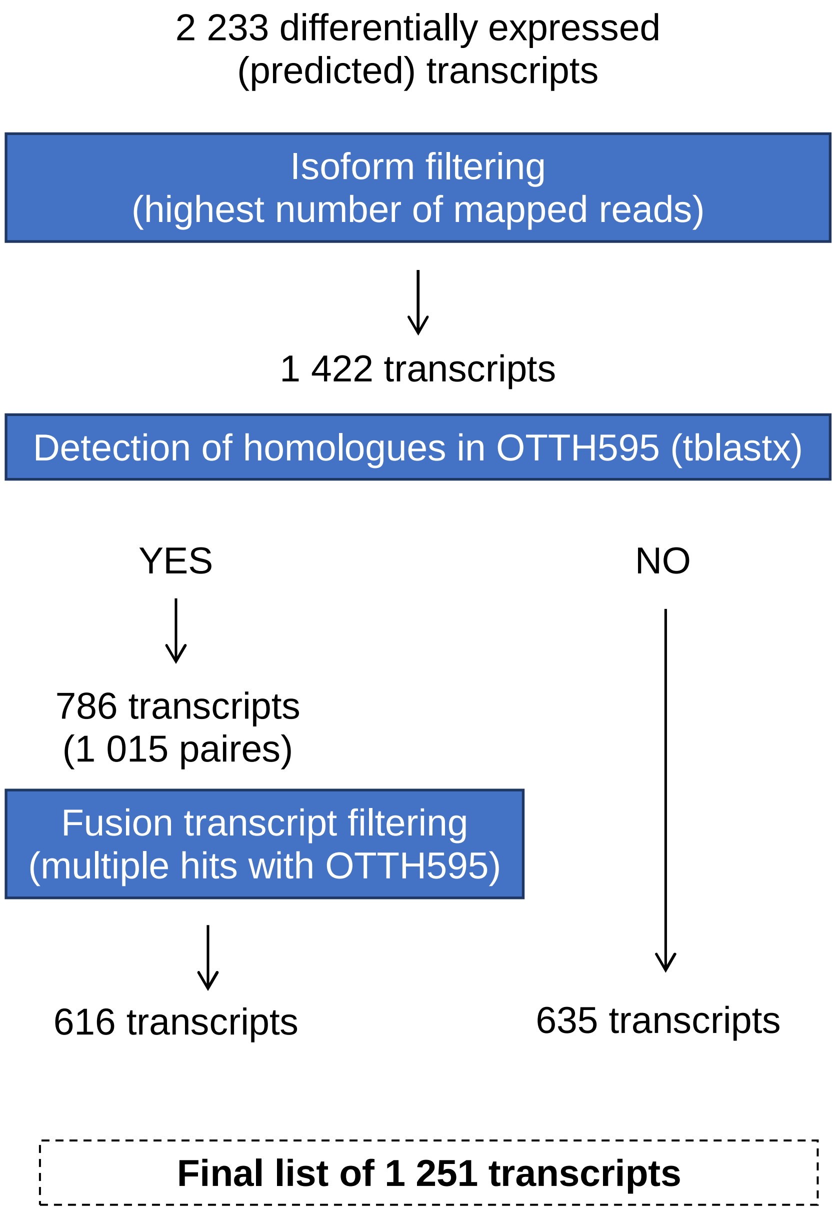

Figure S4 (continued)

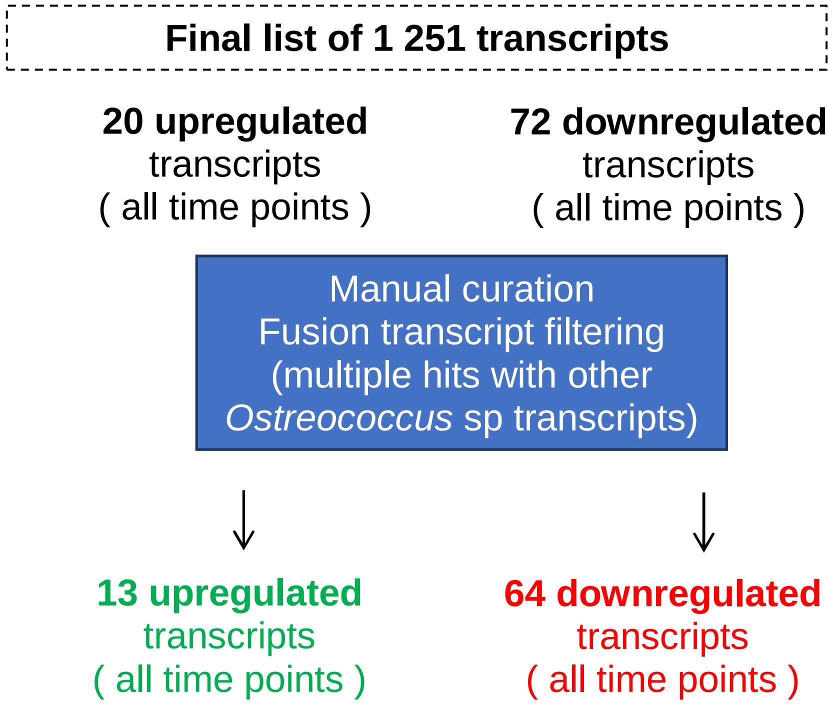

Supplementary Figure 4: Overview of the bioinformatics protocol used to analyze RNAseq data in RCC802. A total of 48 FASTQ files were obtained after RNA sequencing. Reads sequences were filtered with the Trimmomatic program in version 0.36 using a sliding window size of 4 and a minimum quality of 20, cut bases off the start and the end of the read if below a quality of 3, remove read if its size was shorter than 50 bases. After filtration, more than 300 million reads were obtained. Reads from all samples were pulled in order to produce a generic transcriptome that integrates transcripts from all sequenced condition. Assembly was performed using the TRINITY program in version 2.1.1 (Grabherr *et al.*, 2011) with the default parameters and the paired-end method. They comprised more than 300 million reads. In a first step, they were all used for transcriptome assembly using the TRINITY program. 16,527 predicted transcripts were obtained and used for expression quantification applying 1) the BOWTIE program (original read mapping and the predicted transcripts) and 2) the BEDTOOLS program to count the number read aligned on each transcript. DEseq program was applied to identify 2233 transcripts with significant differential expression (comparing -Fe and +Fe samples). Sequence comparisons were finally performed with TBLASTX program to filter transcript isoforms and fusion transcripts. This allowed defining a final list of 1,251 transcripts for which differential expression was observed in at least one time point (see also Figure 6A). 20 and 72 transcripts are respectively up- or downregulated in all conditions. They were manually controlled and functionally annotated based on BLAST searches and database interrogations (ORCAE databases). After this final step, a list of 64 downregulated and 13 upregulated genes was established (see Supplementary data file 1).

Description of supplementary data files

Supplementary data File 1: List of 1251 RCC802 transcripts differentially expressed between iron depleted and iron replete conditions (Excell file)

TRINITY transcript : transcript name (same as in Additional file S1)
Length (nt): Transcript length (nucleotides).
Log FC T1: LogFC (-Fe/+Fe), time 1 (3h)
Pval1: Deseq Pvalue, time1 (3h)
Log FC T2: LogFC (-Fe/+Fe), time 2 (9h)
Pval2: Pvalue, time2 (9h)
Log FC T3: LogFC (-Fe/+Fe), time 3 (15h)
Pval3: Pvalue, time1(15 h)
Log FC T4: LogFC (-Fe/+Fe), time 4 (22h)
Pval4: Pvalue, time 4 (22h)
# UP : Number of time points where logFC>1
# DOWN : Number of time points where logFC<-1
CDS in O. tauri with significant tBLASTX hits (a (at least 70% amino-acids identity, less than 20 mismatches, overlap of 80%).
Description (ORCAE database) : Description in ORCAE Database http://bioinformatics.psb.ugent.be/orcae/overview/OsttaV2)
Protein domains (ORCAE database) : Protein domains of O. tauri http://bioinformatics.psb.ugent.be/orcae/overview/OsttaV2

REFERENCES

Eilers PHC, Peeters JCH. (1988). A model for the relationship between light intensity and the rate of photosynthesis in phytoplankton. *Ecol Modell* **42**:199–215.

Genty B, Briantais J-M, Baker NR. (1989). The relationship between the quantum yield of photosynthetic electron transport and quenching of chlorophyll fluorescence. *Biochim Biophys Acta - Gen Subj* **990**:87–92.

Grabherr MG, Haas BJ, Yassour M, Levin JZ, Thompson DA, Amit I, *et al.* (2011). Full-length transcriptome assembly from RNA-Seq data without a reference genome. *Nat Biotech* **29**:644–652.

Six C, Worden AZ, Rodríguez F, Moreau H, Partensky F. (2005). New insights into the nature and phylogeny of prasinophyte antenna proteins: Ostreococcus tauri, a case study. *Mol Biol Evol* **22**:2217–30.

Zapata M, Rodríguez F, Garrido JL. (2000). Separation of chlorophylls and carotenoids from marine phytoplankton: A new HPLC method using a reversed phase C8 column and pyridine-containing mobile phases. *Mar Ecol Prog Ser* **195**:29–45.
